# Network Pharmacology-Based Study on the Mechanism of Gegen Qinlian Decoction against Colorectal Cancer

**DOI:** 10.1155/2020/8897879

**Published:** 2020-11-26

**Authors:** Qiaowei Fan, Lin Guo, Jingming Guan, Jing Chen, Yujing Fan, Zhendong Chen, Hulun Li

**Affiliations:** ^1^Department of Gastroenterology and Hepatology, The Second Affiliated Hospital of Harbin Medical University, Harbin 150000, China; ^2^Department of Nuclear Medicine, The Second Affiliated Hospital of Harbin Medical University, Harbin 150000, China; ^3^Department of Neurobiology, Harbin Medical University, Harbin 150000, China

## Abstract

**Purpose:**

Gegen Qinlian decoction (GQD) has been used to treat gastrointestinal diseases, such as diarrhea and ulcerative colitis (UC). A recent study demonstrated that GQD enhanced the effect of PD-1 blockade in colorectal cancer (CRC). This study used network pharmacology analysis to investigate the mechanisms of GQD as a potential therapeutic approach against CRC.

**Materials and Methods:**

Bioactive chemical ingredients (BCIs) of GQD were collected from the Traditional Chinese Medicine Systems Pharmacology (TCMSP) database. CRC-specific genes were obtained using the gene expression profile GSE110224 from the Gene Expression Omnibus (GEO) database. Target genes related to BCIs of GQD were then screened out. The GQD-CRC ingredient-target pharmacology network was constructed and visualized using Cytoscape software. A protein-protein interaction (PPI) network was subsequently constructed and analyzed with BisoGenet and CytoNCA plug-in in Cytoscape. Gene Ontology (GO) functional and the Kyoto Encyclopaedia of Genes and Genomes (KEGG) pathway enrichment analysis for target genes were then performed using the *R* package of clusterProfiler.

**Results:**

One hundred and eighteen BCIs were determined to be effective on CRC, including quercetin, wogonin, and baicalein. Twenty corresponding target genes were screened out including PTGS2, CCNB1, and SPP1. Among these genes, CCNB1 and SPP1 were identified as crucial to the PPI network. A total of 212 GO terms and 6 KEGG pathways were enriched for target genes. Functional analysis indicated that these targets were closely related to pathophysiological processes and pathways such as biosynthetic and metabolic processes of prostaglandins and prostanoids, cytokine and chemokine activities, and the IL-17, TNF, Toll-like receptor, and nuclear factor-kappa B (NF-*κ*B) signaling pathways.

**Conclusion:**

The study elucidated the “multiingredient, multitarget, and multipathway” mechanisms of GQD against CRC from a systemic perspective, indicating GQD to be a candidate therapy for CRC treatment.

## 1. Introduction

Colorectal cancer (CRC) is a global health burden and is the third most commonly diagnosed malignancy and the second leading cause of cancer deaths worldwide [1]. It constituted approximately 1.8 million new cases and 900,000 deaths annually, according to estimates from the International Agency for Research on Cancer in 2018 [2]. Despite the progress in the treatment of CRC, effects of current therapies including surgery, radiotherapy, chemotherapy, and targeted therapy are still unsatisfactory, especially for patients with metastatic lesions. Therefore, innovative therapeutic agents are needed, which are more effective and less toxic.

Traditional Chinese medicine (TCM) has been widely used in China for millenniums. It has been proved highly effective for a wide range of diseases. During the fight against infectious pneumonia caused by the 2019 novel coronavirus (2019-nCoV), TCM has made vast contributions to the prevention, treatment, and rehabilitation of coronavirus disease 2019 (COVID-19) among Chinese population [3]. This highlights the great value of TCM in the treatment of complicated diseases, especially those with poor response to Western medicine alone.

Gegen Qinlian decoction (GQD) is a well-known TCM formula originally described in the “Treatise on Exogenous Febrile Disease (“Shang Han Lun” in Chinese).” GQD had been used in China for approximately 2,000 years, most commonly for the treatment of gastrointestinal diseases, such as infectious diarrhea. GQD is composed of four herbal components, Radix Puerariae (“Gegen” in Chinese), Scutellariae Radix (“Huangqin” in Chinese), Coptidis Rhizoma (“Huanglian” in Chinese), and licorice (“Gancao” in Chinese). In recent years, studies have shown promising therapeutic effects of GQD in various diseases. A meta-analysis showed that GQD used alone or in combination with Western medicine might have potential benefits in curing ulcerative colitis (UC). UC can lead to the accumulation of high levels of proinflammatory cytokines within the colonic mucosa, resulting in dysplastic lesions and CRC [4, 5]. GQD has been shown to maintain colonic mucosal homeostasis in ulcerative colitis via bidirectionally regulating Notch signaling [6]. GQD also attenuated high-fat diet-induced steatohepatitis via modulation of the gut microbiome and reduced nonalcoholic steatohepatitis-associated liver injuries [7, 8]. In the field of cancer research, GQD has been proved to inhibit the expansion and neoangiogenesis of renal carcinoma by suppressing matrix metalloproteinase-2 [9]. Moreover, GQD was found to enhance the effect of PD-1 blockade in CRC by remodeling the gut microbiota and the tumor microenvironment [10]. These studies suggest potential for the use of GQD in the treatment of CRC. However, more preclinical evidence is needed. It is difficult to illustrate the complex anticancer mechanisms of GQD due to its “multi-”component and “multi-”target characteristics.

**Figure 1:**
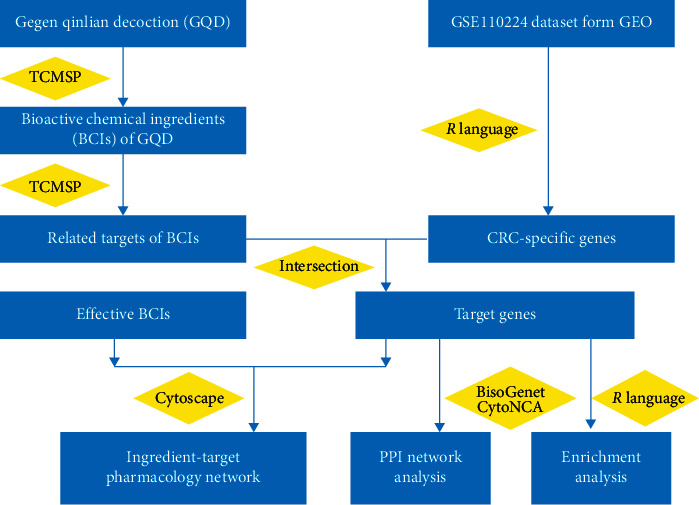
The flowchart of the analysis procedures of the study. Abbreviations: GQD, Gegen Qinlian decoction; BCI, bioactive chemical ingredient; GEO, Gene Expression Omnibus; CRC, colorectal cancer; PPI, protein-protein interaction; TCMSP, Traditional Chinese Medicine Systems Pharmacology.

Network pharmacology, first proposed by Andrew L Hopkins, integrates a series of disciplines including pharmacology, bioinformatics, chemoinformatics, and systems biology. It offers a new framework for drug design and drug-target relationship prediction and enables unknown mechanisms of drug action to be inferred [11]. The history of “TCM network pharmacology” dates back to 1999, when Li proposed a possible relationship between TCM syndrome and molecular networks [12]. Since then, numerous studies have been conducted to support the concept and practice of TCM network pharmacology [13]. The TCM network pharmacology approach provides a new research paradigm for the discovery of bioactive compounds and elucidation of the mechanisms of herbal formulas [13]. TCM has also been successfully used to identify active compounds and elucidate mechanisms of GQD in the treatment of diseases such as type 2 diabetes and rotavirus enteritis [14, 15]. In the present study, we used a network pharmacology-based approach to investigate the potential mechanisms of how GQD exerts its anticancer effects on CRC. First, CRC-specific genes and bioactive chemical ingredients (BCIs) of GQD were obtained from the Gene Expression Omnibus (GEO) and the Traditional Chinese Medicine Systems Pharmacology Database (TCMSP), respectively. Then, CRC-specific genes related to the BCIs of GQD were screened by chemical-target interaction analysis. A pharmacological network and a protein-protein interaction (PPI) network were subsequently constructed to provide a comprehensive overview of the anti-CRC pharmacological action of GQD. Gene Ontology (GO) functional and Kyoto Encyclopaedia of Genes and Genomes (KEGG) pathway enrichment analyses were finally conducted for target genes in the pharmacological network to reveal their functional implications during the anticancer process. The flowchart of the analysis procedures of our study is shown in Figure 1.

## 2. Materials and Methods

### 2.1. CRC-Specific Genes

The microarray expression profile dataset GSE110224 was downloaded from the National Center for Biotechnology Information (NCBI) GEO (http://www.ncbi.nlm.nih.gov/geo/) database, which is based on the GPL570 [HG-U133_Plus_2] Affymetrix Human Genome U133 Plus 2.0 Array platform. The dataset contains 34 samples, including 17 primary colorectal cancer tissue samples and 17 matched normal tissue samples. The raw data were first preprocessed using the Affy package in *R* [16]. Then, the data were converted into expression measures, and background correction, quartile data normalization, and probe summarization were performed using the robust multiarray average (RMA) algorithm in *R* [17]. The paired *t*-test based on the Linear Models for Microarray data (LIMMA) package in *R* was used to identify differentially expressed genes (DEGs) between CRC and normal samples [18]. The DEGs with an adjusted *P* value < 0.05 and a |log_2_ fold-change (log_2_ FC)|≥1 were considered significant and defined as CRC-specific genes.

### 2.2. BCIs of GQD

Chemical compounds were obtained from the TCMSP database (https://tcmspw.com/) [19]. BCIs were screened out according to predicted oral bioavailability (OB) and drug-likeness (DL) values and reserved if OB ≥ 30% and DL ≥ 0.18, which were the suggested drug screening criteria by the TCMSP database.

### 2.3. CRC-Specific Genes Related to BCIs of GQD

All related targets of BCIs from GQD were obtained using TCMSP. After intersection with CRC-specific genes, only genes that were both GQD-related targets and CRC-specific genes were preserved for further analysis. Only BCIs whose targets were CRC-specific genes were used for pharmacological network construction.

### 2.4. Construction of the Pharmacological Network

Based on the data generated by previous steps, a pharmacological network was constructed to illustrate the anti-CRC regulation mechanism between BCIs of GQD and their specific targets. The network was visualized using Cytoscape software (https://cytoscape.org/) [20].

### 2.5. Construction and Analysis of the Protein-Protein Interaction Network

To retrieve all the possible interactions among target genes in the pharmacological network, a PPI network was constructed using the BisoGenet plug-in in Cytoscape [21]. Subsequently, CytoNCA, a Cytoscape plug-in for network centrality analysis, was used to identify crucial genes in the network [22]. Genes with the top 30% highest degree centrality (DC) values were first selected for subnetwork construction using CytoNCA. Then, genes with the top 30% highest betweenness centrality (BC) values in the subnetwork were identified as crucial genes and formed the core network.

### 2.6. Enrichment Analysis for Target Genes and Target-Pathway Network Construction

GO functional enrichment analysis was performed for target genes in three categories: biological process (BP), cellular component (CC), and molecular function (MF) [23]. Both GO functional and KEGG pathway enrichment analysis for target genes were performed using the *R* package of clusterProfiler [24,25]. Benjamini–Hochberg correction was performed for multiple testing, and adjusted *P* value ＜0.05 was set as the threshold. A target-pathway network was then constructed in Cytoscape to visualize the relationships between target genes and KEGG pathways.

## 3. Results

### 3.1. CRC-Specific Genes

Based on the cutoff criteria, a total of 533 DEGs (including 235 upregulated and 298 downregulated genes) were identified between CRC tissues and normal tissues. The top 10 significantly upregulated and downregulated DEGs are listed in Table 1.

### 3.2. BCIs of GQD

Using the TCMSP database, 489 compounds were retrieved: 18 in Gegen, 143 in Huangqin, 48 in Huanglian, and 280 in Gancao. After filtering with the criteria of OB ≥ 30% and DL ≥ 0.18, 146 bioactive components from GQD were collected. These included 4, 36, 14, and 92 from Gegen, Huangqin, Huanglian, and Gancao, respectively. After excluding duplicates, 140 BCIs were selected for further analysis (as shown in Supplementary Table S1).

### 3.3. CRC-Specific Genes Related to BCIs of GQD

A total of 240 BCI-related targets were screened out. After intersecting the 240 BCI-related targets with 533 CRC-specific genes, 20 genes were collected as CRC-specific GQD-target genes. After excluding BCIs whose targets were not CRC-specific, 118 effective BCIs were finally used for pharmacological network construction.

### 3.4. The Pharmacological Network

**Figure 2:**
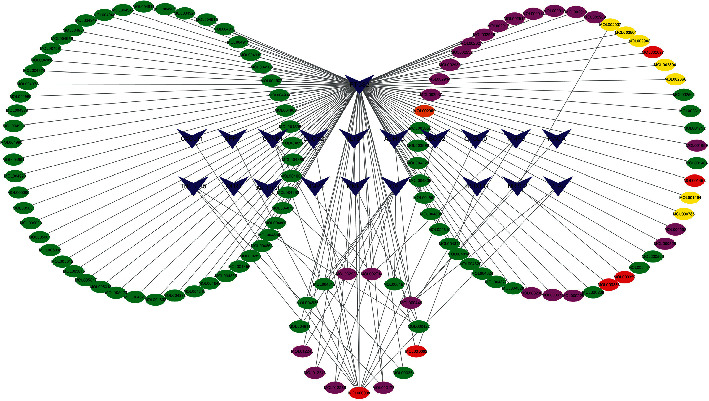
GQD-CRC ingredient-target pharmacology network. Ellipses represent the BCIs of GQD. BCIs of Gancao, Gegen, Huangqin, and Huanglian are painted in green, brown, purple, and yellow, respectively. Shared BCIs of multiple medicines are painted in red. *V*-shaped polygons in blue represent the target genes of BCIs. The regulation relationships between BCIs and their targets are displayed as lines in figure. Abbreviations: BCI, bioactive chemical ingredient; GQD, Gegen Qinlian decoction; CRC, colorectal cancer.

One hundred and eighteen BCIs of GQD together with 20 CRC-specific GQD-target genes were introduced into Cytoscape to create the pharmacological network (Figure 2). BCIs are displayed as ellipse nodes in the network, and BCIs from different herbs are painted in different colors. BCIs from Gancao, Gegen, Huangqin, and Huanglian are painted in green, brown, purple, and yellow, respectively. Shared BCIs of multiple medicines are painted in red. One hundred and three BCIs with only one target are distributed in two larger circles in the upper half of the figure. Fifteen BCIs with multiple targets are distributed in the smaller circle at the bottom of the figure, such as MOL000098 (quercetin), MOL000173 (wogonin), MOL000354 (isorhamnetin), MOL000392 (formononetin), and MOL000422 (kaempferol). Information from 15 BCIs with multiple targets is listed in Table 2. Twenty CRC-specific GQD-target genes are displayed as *V*-shaped polygons in blue, including PTGS2, OLR1, NR3C2, HSD3B2, TNFSF15, MMP1, MMP3, MMP9, AKR1C3, CA2, PLAU, IL1B, DUOX2, CCNB1, ABCG2, CXCL11, CXCL10, SPP1, ADH1C, and MAOA. Information from 20 CRC-specific GQD-target genes, including full name, log_2_FC, adjusted *P* value, and aliases, is shown in Table 3. The regulation relationships between BCIs and their targets are displayed as lines in figure. As the most important gene, PTGS2 is targeted by 116 BCIs and is emphasized centrally in the upper half of the figure.

### 3.5. Construction and Topological Analysis of the PPI Network

**Figure 3:**
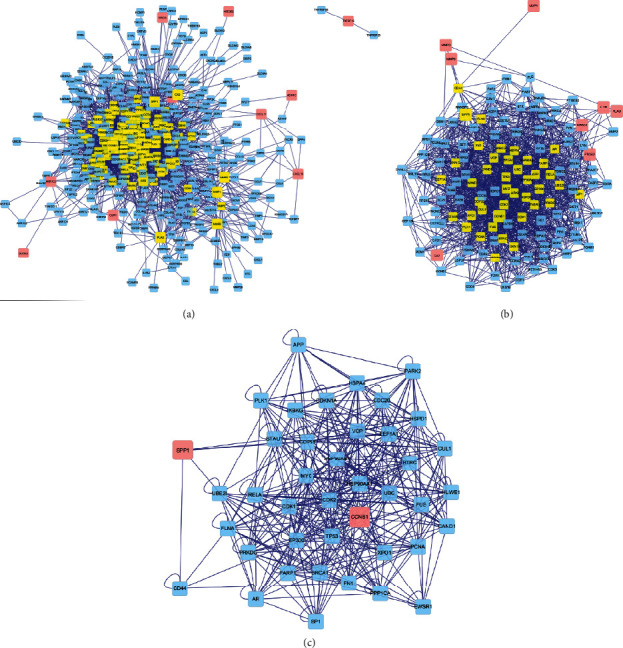
Construction and topological analysis of the PPI network. (a) The PPI network of GQD-CRC target genes generated using BisoGenet, which is comprised of 446 nodes and 3518 edges. Pink nodes represent the CRC-specific GQD-target genes from the pharmacological network. Blue nodes stand for the interacting proteins generated by BisoGenet. The subnetwork with the top 30% highest DC value genes is emphasized in yellow and shown in b. (b) The subnetwork after DC filtration, which is comprised of 130 nodes and 1619 edges. The core network with the top 30% highest BC value genes is emphasized in yellow and shown in c. (c) The core network after BC filtration, which is comprised of 41 nodes and 379 edges. Abbreviations: PPI, protein-protein interaction; GQD, Gegen Qinlian decoction; CRC, colorectal cancer; DC, degree centrality; BC, betweenness centrality.

A PPI network comprising 446 nodes and 3,518 edges was generated using BisoGenet (Figure 3(a)). After DC calculation, 130 nodes together with 1,619 edges were selected to form the subnetwork (Figure 3(b)). Ten target genes including CCNB1, SPP1, MMP9, NR3C2, PLAU, MMP3, PTGS2, CA2, MMP1, and IL1B ranked as the top 30% after DC evaluation and were integrated into the subnetwork, with the degree of 140, 90, 38, 32, 26, 26, 25, 19, 18, and 17, respectively. After BC calculation, 41 nodes and 379 edges were further selected for core network construction (Figure 3(c)). Two target genes, CCNB1 and SPP1, gained the top 30% highest BC values and were finally identified as crucial genes.

### 3.6. Enrichment Analysis for Target Genes and Target-Pathway Network Construction

**Figure 4:**
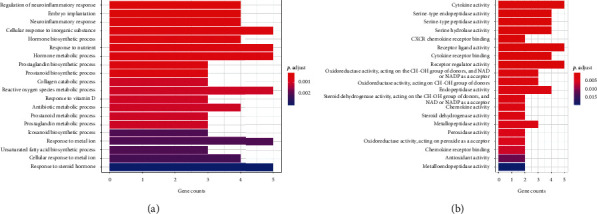
Barplot of GO functional enrichment analysis. (a) The top 20 notable GO-BP terms enriched by target genes in the pharmacological network. (b) The top 20 notable GO-MF terms enriched by target genes in the pharmacological network. Each bar represents a GO term on the vertical axis. The number of genes enriched in each term is recorded on the horizontal axis. Color of each bar represents the adjusted *p* value of each GO term. More red the color of the term is, smaller its adjusted *p* value is. Abbreviations: GO, Gene Ontology; BP, biological process; MF, molecular function.

A total of 171 BP terms and 40 MF terms were enriched for the 20 target genes. The complete BP and MF term lists are shown in Supplementary Tables S2 and S3. Only one CC term (GO: 0070820∼tertiary granule, enriched by OLR1, PLAU, and MMP9 with an adjusted *P* value of 0.038) was enriched under the threshold. The top 20 BP and MF terms are shown in Figures 4(a) and 4(b). Each bar in Figure 4 represents a GO term, plotted by the number of genes enriched in the term on the horizontal axis. The color of each term represents its adjusted *P* value. The more red the color of the term, the smaller its adjusted *P* value.

**Figure 5:**
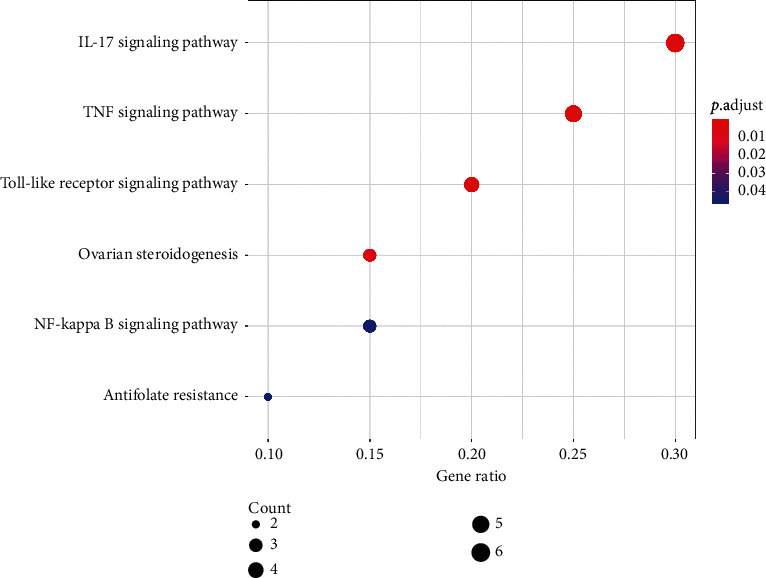
Bubble graph of KEGG pathway enrichment analysis. Each bubble represents a KEGG pathway on the vertical axis. The gene ratio is recorded on the horizontal axis. The size of each bubble indicates the number of genes enriched in each KEGG pathway. Larger the bubble is, more number of genes is involved in the pathway. Color of each bubble represents the adjusted *P* value of each KEGG pathway. More red the color of the bubble is, smaller its adjusted *P* value is. Abbreviation: KEGG, Kyoto Encyclopaedia of Genes and Genomes.

**Figure 6:**
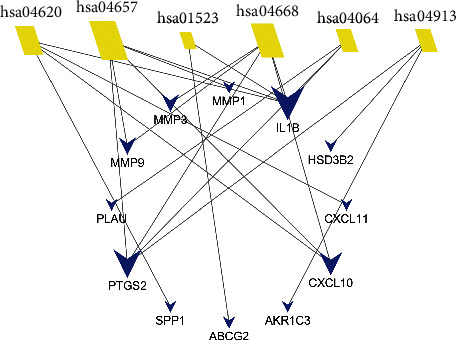
Target-pathway network. Blue *V*-shaped polygons represent target genes. Yellow parallelograms represent KEGG pathways logoed by its identifier number in KEGG database. Larger the size of the *V* polygon is, more KEGG pathways the target gene is involved in. Larger the size of the parallelogram is, more number of target genes the KEGG pathway contains. Abbreviation: KEGG, Kyoto Encyclopaedia of Genes and Genomes.

Six KEGG pathways were enriched for target genes, and these are listed in Table 4. The bubble graph of KEGG pathways is shown in Figure 5, with gene ratio on the horizontal axis. The size of each bubble indicates the number of genes enriched in each KEGG pathway. The larger the bubble, the greater the number of genes involved in the pathway. As in the GO barplot, the color of each bubble in Figure 5 represents the adjusted *P* value of each KEGG pathway. The more red the color of the bubble, the smaller its adjusted *P* value. The target-pathway network is displayed in Figure 6. Target genes and KEGG pathways are visualized as *V*-shaped polygons and parallelograms, respectively. The larger the size of the *V* polygon, the more KEGG pathways the target gene is involved in. Similarly, the larger the size of the parallelogram, the greater the number of target genes the KEGG pathway contains.

## 4. Discussion

Colorectal carcinogenesis is a complex and consecutive progression. It involves a multiscale and systemic framework integrating genetic, proteomic, and metabolic networks from responses to DNA damage, gene mutations, population dynamics, inflammation, and metabolism-immune balance [26–28]. The evolution of the genomic landscape through novel sequencing techniques has uncovered major clues into the key mechanisms of CRC. Medicines have been designed to target specific genetic keypoints to block the progression of the disease [29]. However, survival of CRC patients remains unsatisfactory due to the complex crosstalk among these alterations. TCM has unique benefits such as being naturally sourced, multitargeted, and a holistic concept. It has advantages in treating complicated diseases, especially those with a poor response to Western medicine alone. In the present study, we used a network pharmacology-based approach to reveal the pharmacological effects of GQD on CRC, which might provide novel therapeutic strategies for better treatment of CRC.

Network pharmacology analysis showed that 20 target genes were regulated by GQD in CRC, including PTGS2, CCNB1, SPP1, PLAU, MAOA, OLR1, NR3C2, HSD3B2, TNFSF15, AKR1C3, CA2, MMP1, MMP3, MMP9, IL1B, DUOX2, ABCG2, CXCL11, CXCL10, and ADH1C. Most of these have been reportedly associated with CRC.

PTGS2 (prostaglandin G/H synthase 2, also known as PGHS-2; COX-2) is one of the most important genes in the pharmacological network. Many studies have demonstrated that CRC is closely related to PTGS2. Studies have found PTGS2 to be overexpressed in CRC tissues [30], which is consistent with results in the present study (Table 3). The elevation of PTGS2 predicts poor prognosis in colon cancer [31]. PTGS2 produces the inflammatory mediator prostaglandin E2 (PGE2), which is suggested to promote the development and progression of CRC [32–34]. Furthermore, epidemiological evidence indicates that the regular use of aspirin (a PTGS2 inhibitor) reduces the risk of CRC [35]. In the present study, PTGS2 was targeted by as many as 116 BCIs in GQD, indicating it may possess a potentially important PTGS2-related anti-CRC mechanism.

Reduction of NR3C2 (also known as MR) expression was found to be a potential early event involved in CRC progression [36]. Expression of AKR1C3 may be used for the prediction of lymph node metastasis in CRC [37]. High mRNA expression of DUOX2 was significantly associated with better overall survival of CRC patients [38]. ABCG2 was shown to play a potential protective role in CRC by inhibiting the NF-*κ*B signaling pathway to relieve oxidative stress and decrease the inflammatory response [39]. Downregulation of CXCL11 inhibited cell growth and epithelial-mesenchymal transition in CRC [40]. The crucial MMP1, MMP3, and MMP9 genes in the pharmacology network are all matrix metalloproteinase family members. Increasing evidence has demonstrated their oncogenic significance in CRC carcinogenesis [41–43].

After PPI network analysis, CCNB1 and SPP1 were identified as crucial genes of the highest degree. Cyclin B1 (CCNB1) is a well-known gene involved in mitosis. It produces a complex with cyclin-dependent kinase 1 (CDK1), which is necessary for proper control of the G2/M transition phase of the cell cycle [44, 45]. Previous study indicated that CCNB1 is overexpressed in CRC tissues, and inhibition of CCNB1 suppressed the proliferation of CRC cells in vitro and tumorigenicity in vivo [46]. SPP1, also known as OPN, has been shown to regulate multiple functions contributing to CRC progression [47]. Upregulation of SPP1 promoted CRC cell proliferation in vitro and tumor growth in vivo [47]. It also promoted metastasis in CRC by activating the EMT pathway and was associated with poor survival outcomes in CRC [48, 49].

Fifteen BCIs were correlated with multiple target genes in the pharmacological network. Some of these have already been shown to exert anti-CRC properties. Quercetin, a dietary flavonoid, was reported to induce human colon cancer cell apoptosis by inhibiting the NF-*κ*B pathway and inducing apoptosis in KRAS-mutant CRC cells via JNK signaling pathways [50, 51]. Wogonin, a naturally occurring monoflavonoid, induced antiproliferation and G1 arrest via the Wnt/*β*-catenin signaling pathway in CRC cells [52]. Baicalein was shown to inhibit the proliferation and migration of CRC cells [53–55]. Oroxylin A was reported to suppress the growth and development of CRC via reprogramming of HIF1*α*-modulated fatty acid metabolism [56]. These findings all suggest the promising potential of GQD in the treatment of CRC.

Based on GO enrichment analysis, BP terms enriched by target genes were mainly concentrated in response to various materials and biochemical processes of different substances. Response to inorganic substance (GO: 0071241), nutrient (GO: 0007584), vitamin D (GO: 0033280), and metal ion (GO: 0010038) are all pivotal in cellular metabolism. Biosynthetic and metabolic processes of prostaglandins and prostanoids (including GO:0006693, GO:0046457, GO:0006692, and GO:0001516) are also important in colorectal tumorigenesis, since prostaglandins and prostanoids have been implicated in various pathological processes in CRC [57–60]. Some enriched MF terms are associated with inflammation, such as GO:0005125: cytokine activity, GO:0005126: cytokine receptor binding, GO:0042379: chemokine receptor binding, GO:0008009: chemokine activity, and GO:0045236: CXCR chemokine receptor binding. Inflammation, a hallmark of CRC, has been discussed in many publications. Cytokines such as interleukin-6 (IL-6), interleukin-7 (IL-7), interleukin-10 (IL-10), and interleukin-17 (IL-17) have been shown to be involved in the development of CRC [61–64]. Chemokines direct leukocyte recruitment and migration under inflammatory conditions [65, 66]. The expression of many chemokines, such as CCL2–4, CXCL1, CXCL5, and CXCL8–10, was reportedly elevated in the CRC microenvironment compared to normal tissues [67, 68].

KEGG enrichment analysis showed that the pharmacological effects of GQD on CRC are closely related to well-known tumor-associated pathways, including the IL-17, tumor necrosis factor (TNF), Toll-like receptor, and NF-*κ*B signaling pathways. Numerous studies have highlighted the important role of the IL-17 signaling pathway in the tumorigenesis, angiogenesis, and metastasis of CRC. Interleukin-17 (IL-17), a proinflammatory cytokine, was significantly upregulated in CRC tissues [69]. IL-17 can promote CRC tumorigenesis by stimulating the production and recruitment of myeloid-derived suppressor cells (MDSCs) [70]. It promotes angiogenesis via stimulating VEGF production in CRC cells [71]. Additionally, IL-17 stimulates the production of PGE2, MMP9, and MMP13, which are involved in the migration of CRC cells [72–74].

TNF-*α* is one of the most important cell signaling proteins involved in cell growth, differentiation, and apoptosis [75, 76]. It plays a pivotal role in proliferation, angiogenesis, and metastasis in CRC [77]. Serum TNF-*α* was demonstrated to contribute to CRC susceptibility, and anti-TNF therapy has been considered for CRC treatment [78].

A large body of data demonstrates an important relationship between the Toll-like receptor (TLR) signaling pathway and CRC. The TLR signaling pathway exerts a fundamental role in colorectal epithelium hemostasis and in activating the innate and adaptive immune responses [79]. Activation of the TLR signaling pathway leads to activation of downstream signaling pathways and recruitment of transcription factors such as NF-*κ*B, interferon regulatory factor- (IRF-) 3, AP-1, PI3K/Akt kinases, the mitogen-activated protein kinase (MAPK), and the subsequent generation of cytokines and chemokines [80, 81]. The TLR2 and TLR4 agonists HMGB1 and S100A9 have been proposed as potential biomarkers for CRC [82, 83]. Several TLR-based therapeutic agents have been developed for targeting this pathway and are currently used in clinical trials in patients with CRC [84–86].

The NF-*κ*B signaling pathway is a key regulator of CRC cell proliferation, apoptosis, inflammation, angiogenesis metastasis, and drug resistance [87]. Constitutive NF-*κ*B activation was observed in CRC cell lines and human CRCs [88, 89]. It promotes the proliferation of cancer cells and rescues cancer cells from cell death [90]. Studies suggest that inhibition of the NF-*κ*B pathway can sensitize CRC cells to chemotherapy and radiotherapy, providing more effective strategies for cancer treatment [91, 92].

These findings suggest that the molecular mechanisms of GQD against CRC are closely related to these key target genes, biochemical processes, and important signaling pathways. However, detailed experiments are still needed to confirm these findings.

## 5. Conclusions

In conclusion, this study is the first to reveal the pharmacological effects of GQD against CRC via network pharmacology analysis. A total of 118 BCIs from GQD were identified, and 20 corresponding genes including PTGS2, NR3C2, CXCL11, CCNB1, and SPP1 were demonstrated to be key targets for GQD in CRC. GO functional and KEGG pathway enrichment analysis indicated that the molecular mechanisms of GQD in CRC were closely related to important biochemical processes and signaling pathways, such as biosynthetic and metabolic processes of prostaglandins and prostanoids, cytokine and chemokine activities, the IL-17 signaling pathway, the TNF signaling pathway, the Toll-like receptor signaling pathway, and the NF-*κ*B signaling pathway. The study provides a research basis for further studies of GQD in the treatment of CRC.

## Figures and Tables

**Table 1:** The identified top 10 upregulated and downregulated DEGs between CRC tissue samples and normal tissue samples.

Upregulated DEGs			Downregulated DEGs		
Gene name	Log_2_FC	Adjusted *P* value	Gene name	Log_2_FC	Adjusted *P* value
MMP3	4.1101	1.41*E* − 03	CLCA4	−4.4741	4.45*E* − 03
REG1A	3.8436	5.89*E* − 03	MS4A12	−4.4636	4.03*E* − 03
FOXQ1	3.5684	1.79*E* − 03	AQP8	−4.3366	1.91*E* − 03
CXCL11	3.4122	2.12*E* − 03	CA4	−3.6607	2.16*E* − 03
MMP7	3.3618	1.56*E* − 03	GCG	−3.6245	9.42*E* − 03
REG1B	3.3276	2.67*E* − 02	GUCA2A	−3.4325	3.83*E* − 03
MMP1	3.2300	2.86*E* −03	CLDN8	−3.3370	1.14*E* − 02
TCN1	3.2266	3.83*E* − 03	CHP2	−3.2031	1.58*E* − 03
SLC6A14	2.7141	6.57*E* − 03	NXPE4	−3.1622	3.12*E* − 03
CXCL3	2.6473	5.58*E* − 03	MAMDC2	−3.0890	3.21*E* − 03

DEGs, differentially expressed genes; FC, fold change.

**Table 2:** Information of 15 BCIs with multiple targets in the pharmacological network.

Molecule ID	Molecule name	Molecule structure	OB (%)	DL	Source
MOL000098	Quercetin	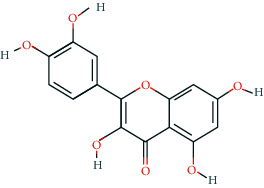	46.43	0.28	Huanglian and Gancao

MOL000173	Wogonin	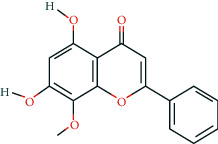	30.68	0.23	Huangqin

MOL000354	Isorhamnetin	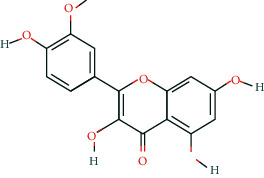	49.6	0.31	Gancao

MOL000392	Formononetin	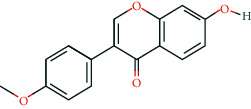	69.67	0.21	Gegen and Gancao

MOL000422	Kaempferol	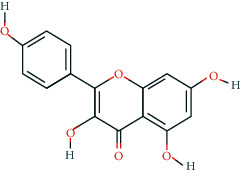	41.88	0.24	Gancao

MOL000449	Stigmasterol	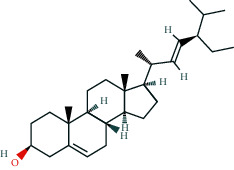	43.83	0.76	Huangqin

MOL000497	Licochalcone a	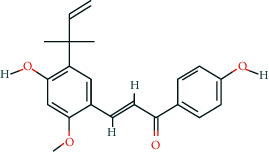	40.79	0.29	Gancao

MOL002714	Baicalein	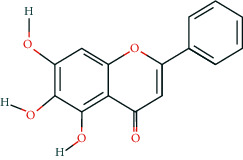	33.52	0.21	Huangqin

MOL002928	Oroxylin a	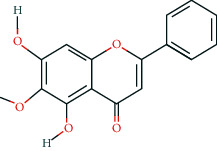	41.37	0.23	Huangqin

MOL004815	(E)-1-(2,4-Dihydroxyphenyl)-3-(2,2-dimethylchromen-6-yl)prop-2-en-1-one	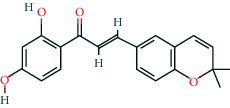	39.62	0.35	Gancao

MOL004835	Glypallichalcone	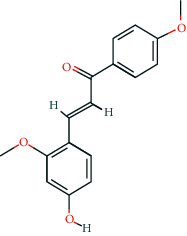	61.6	0.19	Gancao

MOL004841	Licochalcone B	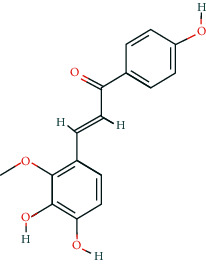	76.76	0.19	Gancao

MOL012245	5,7,4′-Trihydroxy-6-methoxyflavanone	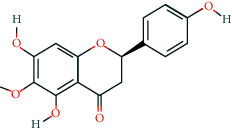	36.63	0.27	Huangqin

MOL012246	5,7,4′-Trihydroxy-8-methoxyflavanone	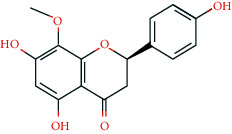	74.24	0.26	Huangqin

MOL012266	Rivularin	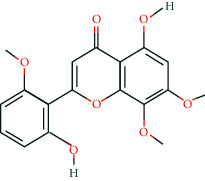	37.94	0.37	Huangqin

BCI, bioactive chemical ingredient; OB, oral bioavailability; DL, drug-likeness.

**Table 3:** Information of 20 target genes in the pharmacological network.

Gene	Full name	Log_2_FC	Adjusted *P* value	Aliases
PTGS2	Prostaglandin G/H synthase 2	1.7769	7.39*E* − 03	PGHS-2; COX-2; PHS-2; PGG/HS; hCox-2; GRIPGHS
OLR1	Oxidized low-density lipoprotein receptor 1	1.9948	4.17*E* − 03	LOX1; LOXIN; SLOX1; CLEC8A; SCARE1
NR3C2	Nuclear receptor subfamily 3 group C member 2	−1.5129	3.92*E* − 03	MR; MCR; MLR; NR3C2VIT
HSD3B2	Hydroxy-delta-5-steroid dehydrogenase, 3 beta- and steroid delta-isomerase 2	−1.1592	8.47*E* − 03	HSDB; HSD3B; SDR11E2
TNFSF15	TNF superfamily member 15	1.0461	1.24*E* − 02	TL1; TL1A; VEGI; TNLG1B; VEGI192A
MMP1	Matrix metallopeptidase 1	3.2300	2.86*E* − 03	CLG; CLGN
AKR1C3	Aldo-keto reductase family 1 member C3	−1.2267	6.79*E* − 03	DD3; DDX; PGFS; HAKRB; HAKRe; HA1753; HSD17B5; hluPGFS
CA2	Carbonic anhydrase 2	−1.7184	9.85*E* − 03	CAC; CAII; Car2; CAII; HEL-76; HEL-S-282
MMP3	Matrix metallopeptidase 3	4.1101	1.41*E* − 03	SL-1; STMY; STR1; CHDS6; MMP3; STMY1
PLAU	Plasminogen activator, urokinase	1.2642	4.83*E* − 03	ATF; QPD; UPA; URK; u-PA; BDPLT5
MMP9	Matrix metallopeptidase 9	1.1245	8.08*E* − 03	GELB; CLG4B; MMP9; MANDP2
IL1B	Interleukin 1 beta	2.1239	2.27*E* − 03	IL-1; IL1F2; IL1beta; IL1-BETA
DUOX2	Dual oxidase 2	1.9328	2.98*E* − 02	TDH6; LNOX2; THOX2; NOXEF2; P138-TOX
CCNB1	Cyclin B1	1.0204	1.50*E* − 02	CCNB
ABCG2	ATP binding cassette subfamily G member 2	−2.9521	2.92*E* − 03	MRX; MXR; ABCP; BCRP; BMDP; MXR1; ABC15; BCRP1; CD338; GOUT1; MXR-1; CDw338; UAQTL1; EST157481
CXCL11	C-X-C motif chemokine ligand 11	3.4122	2.12*E* − 03	IP9; H174; IP-9; b-R1; I-TAC; SCYB11; SCYB9B
CXCL10	C-X-C motif chemokine ligand 10	2.1322	6.06*E* − 03	C7; IFI10; INP10; IP-10; crg-2; mob-1; SCYB10; gIP-10
SPP1	Secreted phosphoprotein 1	1.5076	7.22*E* − 03	OPN; BNSP; BSPI; ETA-1
ADH1C	Alcohol dehydrogenase 1C (class I), gamma polypeptide	−2.9047	7.54*E* − 03	ADH3
MAOA	Monoamine oxidase A	−1.1045	7.88*E* − 03	BRNRS; MAO-a

FC, fold change.

**Table 4:** KEGG pathways enriched by target genes.

ID	Description	Adjusted *P* value
Hsa04657	IL-17 signaling pathway	9.27*E* − 06
Hsa04668	TNF signaling pathway	3.79*E* − 04
Hsa04620	Toll-like receptor signaling pathway	4.21*E* − 03
Hsa04913	Ovarian steroidogenesis	7.08*E* − 03
Hsa04064	NF-kappa B signaling pathway	4.53*E* − 02
hsa01523	Antifolate resistance	4.67*E* − 02

KEGG, Kyoto Encyclopaedia of Genes and Genomes.

## Data Availability

The data used to support the findings of this study are included within the article and supplementary information files.

## Supplementary Materials

Supplementary Materials Supplementary Table S1: information for 140 BCIs of GQD. Supplementary Table S2: the significant GO-BP terms enriched by target genes. Supplementary Table S3: the significant GO-MF terms enriched by target genes.
