# Supplementary material for: Network Pharmacology-Based Study on the Mechanism of Gegen Qinlian Decoction against Colorectal Cancer
Source: Evid Based Complement Alternat Med. 2020 Nov 26;2020:8897879. doi: 10.1155/2020/8897879 (PMC7714584; doi:10.1155/2020/8897879)
Supplement: Supplementary Materials — Supplementary Table S1: information for 140 BCIs of GQD. Supplementary Table S2: the significant GO-BP terms enriched by target genes. Supplementary Table S3: the significant GO-MF terms enriched by target genes. [file 8897879.f1.docx]

**Supplementary Materials**

**TABLE S1:** Information for 140 BCIs of GQD.

| Mol ID | Molecule Name | OB (%) | DL | Source |
| --- | --- | --- | --- | --- |
| MOL002959 | 3'-Methoxydaidzein | 48.57 | 0.24 | Gegen |
| MOL003629 | Daidzein-4,7-diglucoside | 47.27 | 0.67 | Gegen |
| MOL000392 | formononetin | 69.67 | 0.21 | Gegen,Gancao |
| MOL000358 | beta-sitosterol | 36.91 | 0.75 | Gegen,Huangqin |
| MOL000073 | ent-Epicatechin | 48.96 | 0.24 | Huangqin |
| MOL000173 | wogonin | 30.68 | 0.23 | Huangqin |
| MOL000228 | (2R)-7-hydroxy-5-methoxy-2-phenylchroman-4-one | 55.23 | 0.2 | Huangqin |
| MOL000449 | Stigmasterol | 43.83 | 0.76 | Huangqin |
| MOL000525 | Norwogonin | 39.4 | 0.21 | Huangqin |
| MOL000552 | 5,2'-Dihydroxy-6,7,8-trimethoxyflavone | 31.71 | 0.35 | Huangqin |
| MOL001490 | bis[(2S)-2-ethylhexyl] benzene-1,2-dicarboxylate | 43.59 | 0.35 | Huangqin |
| MOL001506 | Supraene | 33.55 | 0.42 | Huangqin |
| MOL001689 | acacetin | 34.97 | 0.24 | Huangqin |
| MOL002714 | baicalein | 33.52 | 0.21 | Huangqin |
| MOL002879 | Diop | 43.59 | 0.39 | Huangqin |
| MOL002908 | 5,8,2'-Trihydroxy-7-methoxyflavone | 37.01 | 0.27 | Huangqin |
| MOL002909 | 5,7,2,5-tetrahydroxy-8,6-dimethoxyflavone | 33.82 | 0.45 | Huangqin |
| MOL002910 | Carthamidin | 41.15 | 0.24 | Huangqin |
| MOL002911 | 2,6,2',4'-tetrahydroxy-6'-methoxychaleone | 69.04 | 0.22 | Huangqin |
| MOL002913 | Dihydrobaicalin_qt | 40.04 | 0.21 | Huangqin |
| MOL002914 | Eriodyctiol (flavanone) | 41.35 | 0.24 | Huangqin |
| MOL002915 | Salvigenin | 49.07 | 0.33 | Huangqin |
| MOL002917 | 5,2',6'-Trihydroxy-7,8-dimethoxyflavone | 45.05 | 0.33 | Huangqin |
| MOL002925 | 5,7,2',6'-Tetrahydroxyflavone | 37.01 | 0.24 | Huangqin |
| MOL002926 | dihydrooroxylin A | 38.72 | 0.23 | Huangqin |
| MOL002927 | Skullcapflavone II | 69.51 | 0.44 | Huangqin |
| MOL002928 | oroxylin a | 41.37 | 0.23 | Huangqin |
| MOL002932 | Panicolin | 76.26 | 0.29 | Huangqin |
| MOL002933 | 5,7,4'-Trihydroxy-8-methoxyflavone | 36.56 | 0.27 | Huangqin |
| MOL002934 | NEOBAICALEIN | 104.34 | 0.44 | Huangqin |
| MOL002937 | DIHYDROOROXYLIN | 66.06 | 0.23 | Huangqin |
| MOL008206 | Moslosooflavone | 44.09 | 0.25 | Huangqin |
| MOL010415 | 11,13-Eicosadienoic acid, methyl ester | 39.28 | 0.23 | Huangqin |
| MOL012245 | 5,7,4'-trihydroxy-6-methoxyflavanone | 36.63 | 0.27 | Huangqin |
| MOL012246 | 5,7,4'-trihydroxy-8-methoxyflavanone | 74.24 | 0.26 | Huangqin |
| MOL012266 | rivularin | 37.94 | 0.37 | Huangqin |
| MOL002897 | epiberberine | 43.09 | 0.78 | Huangqin,Huanglian |
| MOL001458 | coptisine | 30.67 | 0.86 | Huangqin,Huanglian |
| MOL000622 | Magnograndiolide | 63.71 | 0.19 | Huanglian |
| MOL000762 | Palmidin A | 35.36 | 0.65 | Huanglian |
| MOL000785 | palmatine | 64.6 | 0.65 | Huanglian |
| MOL001454 | berberine | 36.86 | 0.78 | Huanglian |
| MOL002668 | Worenine | 45.83 | 0.87 | Huanglian |
| MOL002894 | berberrubine | 35.74 | 0.73 | Huanglian |
| MOL002903 | (R)-Canadine | 55.37 | 0.77 | Huanglian |
| MOL002904 | Berlambine | 36.68 | 0.82 | Huanglian |
| MOL002907 | Corchoroside A_qt | 104.95 | 0.78 | Huanglian |
| MOL008647 | Moupinamide | 86.71 | 0.26 | Huanglian |
| MOL013352 | Obacunone | 43.29 | 0.77 | Huanglian |
| MOL000098 | quercetin | 46.43 | 0.28 | Huanglian,Gancao |
| MOL000211 | Mairin | 55.38 | 0.78 | Gancao |
| MOL000239 | Jaranol | 50.83 | 0.29 | Gancao |
| MOL000354 | isorhamnetin | 49.6 | 0.31 | Gancao |
| MOL000417 | Calycosin | 47.75 | 0.24 | Gancao |
| MOL000422 | kaempferol | 41.88 | 0.24 | Gancao |
| MOL000497 | licochalcone a | 40.79 | 0.29 | Gancao |
| MOL000500 | Vestitol | 74.66 | 0.21 | Gancao |
| MOL001484 | Inermine | 75.18 | 0.54 | Gancao |
| MOL001792 | DFV | 32.76 | 0.18 | Gancao |
| MOL002311 | Glycyrol | 90.78 | 0.67 | Gancao |
| MOL002565 | Medicarpin | 49.22 | 0.34 | Gancao |
| MOL003656 | Lupiwighteone | 51.64 | 0.37 | Gancao |
| MOL003896 | 7-Methoxy-2-methyl isoflavone | 42.56 | 0.2 | Gancao |
| MOL004328 | naringenin | 59.29 | 0.21 | Gancao |
| MOL004805 | (2S)-2-[4-hydroxy-3-(3-methylbut-2-enyl)phenyl]-8,8-dimethyl-2,3-dihydropyrano[2,3-f]chromen-4-one | 31.79 | 0.72 | Gancao |
| MOL004806 | euchrenone | 30.29 | 0.57 | Gancao |
| MOL004808 | glyasperin B | 65.22 | 0.44 | Gancao |
| MOL004810 | glyasperin F | 75.84 | 0.54 | Gancao |
| MOL004811 | Glyasperin C | 45.56 | 0.4 | Gancao |
| MOL004814 | Isotrifoliol | 31.94 | 0.42 | Gancao |
| MOL004815 | (E)-1-(2,4-dihydroxyphenyl)-3-(2,2-dimethylchromen-6-yl)prop-2-en-1-one | 39.62 | 0.35 | Gancao |
| MOL004820 | kanzonols W | 50.48 | 0.52 | Gancao |
| MOL004824 | (2S)-6-(2,4-dihydroxyphenyl)-2-(2-hydroxypropan-2-yl)-4-methoxy-2,3-dihydrofuro[3,2-g]chromen-7-one | 60.25 | 0.63 | Gancao |
| MOL004827 | Semilicoisoflavone B | 48.78 | 0.55 | Gancao |
| MOL004828 | Glepidotin A | 44.72 | 0.35 | Gancao |
| MOL004829 | Glepidotin B | 64.46 | 0.34 | Gancao |
| MOL004833 | Phaseolinisoflavan | 32.01 | 0.45 | Gancao |
| MOL004835 | Glypallichalcone | 61.6 | 0.19 | Gancao |
| MOL004838 | 8-(6-hydroxy-2-benzofuranyl)-2,2-dimethyl-5-chromenol | 58.44 | 0.38 | Gancao |
| MOL004841 | Licochalcone B | 76.76 | 0.19 | Gancao |
| MOL004848 | licochalcone G | 49.25 | 0.32 | Gancao |
| MOL004849 | 3-(2,4-dihydroxyphenyl)-8-(1,1-dimethylprop-2-enyl)-7-hydroxy-5-methoxy-coumarin | 59.62 | 0.43 | Gancao |
| MOL004855 | Licoricone | 63.58 | 0.47 | Gancao |
| MOL004856 | Gancaonin A | 51.08 | 0.4 | Gancao |
| MOL004857 | Gancaonin B | 48.79 | 0.45 | Gancao |
| MOL004860 | licorice glycoside E | 32.89 | 0.27 | Gancao |
| MOL004863 | 3-(3,4-dihydroxyphenyl)-5,7-dihydroxy-8-(3-methylbut-2-enyl)chromone | 66.37 | 0.41 | Gancao |
| MOL004864 | 5,7-dihydroxy-3-(4-methoxyphenyl)-8-(3-methylbut-2-enyl)chromone | 30.49 | 0.41 | Gancao |
| MOL004866 | 2-(3,4-dihydroxyphenyl)-5,7-dihydroxy-6-(3-methylbut-2-enyl)chromone | 44.15 | 0.41 | Gancao |
| MOL004879 | Glycyrin | 52.61 | 0.47 | Gancao |
| MOL004882 | Licocoumarone | 33.21 | 0.36 | Gancao |
| MOL004883 | Licoisoflavone | 41.61 | 0.42 | Gancao |
| MOL004884 | Licoisoflavone B | 38.93 | 0.55 | Gancao |
| MOL004885 | licoisoflavanone | 52.47 | 0.54 | Gancao |
| MOL004891 | shinpterocarpin | 80.3 | 0.73 | Gancao |
| MOL004898 | (E)-3-[3,4-dihydroxy-5-(3-methylbut-2-enyl)phenyl]-1-(2,4-dihydroxyphenyl)prop-2-en-1-one | 46.27 | 0.31 | Gancao |
| MOL004903 | liquiritin | 65.69 | 0.74 | Gancao |
| MOL004904 | licopyranocoumarin | 80.36 | 0.65 | Gancao |
| MOL004905 | 3,22-Dihydroxy-11-oxo-delta(12)-oleanene-27-alpha-methoxycarbonyl-29-oic acid | 34.32 | 0.55 | Gancao |
| MOL004907 | Glyzaglabrin | 61.07 | 0.35 | Gancao |
| MOL004908 | Glabridin | 53.25 | 0.47 | Gancao |
| MOL004910 | Glabranin | 52.9 | 0.31 | Gancao |
| MOL004911 | Glabrene | 46.27 | 0.44 | Gancao |
| MOL004912 | Glabrone | 52.51 | 0.5 | Gancao |
| MOL004913 | 1,3-dihydroxy-9-methoxy-6-benzofurano[3,2-c]chromenone | 48.14 | 0.43 | Gancao |
| MOL004914 | 1,3-dihydroxy-8,9-dimethoxy-6-benzofurano[3,2-c]chromenone | 62.9 | 0.53 | Gancao |
| MOL004915 | Eurycarpin A | 43.28 | 0.37 | Gancao |
| MOL004917 | glycyroside | 37.25 | 0.79 | Gancao |
| MOL004924 | (-)-Medicocarpin | 40.99 | 0.95 | Gancao |
| MOL004935 | Sigmoidin-B | 34.88 | 0.41 | Gancao |
| MOL004941 | (2R)-7-hydroxy-2-(4-hydroxyphenyl)chroman-4-one | 71.12 | 0.18 | Gancao |
| MOL004945 | (2S)-7-hydroxy-2-(4-hydroxyphenyl)-8-(3-methylbut-2-enyl)chroman-4-one | 36.57 | 0.32 | Gancao |
| MOL004948 | Isoglycyrol | 44.7 | 0.84 | Gancao |
| MOL004949 | Isolicoflavonol | 45.17 | 0.42 | Gancao |
| MOL004957 | HMO | 38.37 | 0.21 | Gancao |
| MOL004959 | 1-Methoxyphaseollidin | 69.98 | 0.64 | Gancao |
| MOL004961 | Quercetin der. | 46.45 | 0.33 | Gancao |
| MOL004966 | 3'-Hydroxy-4'-O-Methylglabridin | 43.71 | 0.57 | Gancao |
| MOL004974 | 3'-Methoxyglabridin | 46.16 | 0.57 | Gancao |
| MOL004978 | 2-[(3R)-8,8-dimethyl-3,4-dihydro-2H-pyrano[6,5-f]chromen-3-yl]-5-methoxyphenol | 36.21 | 0.52 | Gancao |
| MOL004980 | Inflacoumarin A | 39.71 | 0.33 | Gancao |
| MOL004985 | icos-5-enoic acid | 30.7 | 0.2 | Gancao |
| MOL004988 | Kanzonol F | 32.47 | 0.89 | Gancao |
| MOL004989 | 6-prenylated eriodictyol | 39.22 | 0.41 | Gancao |
| MOL004990 | 7,2',4'-trihydroxy－5-methoxy-3－arylcoumarin | 83.71 | 0.27 | Gancao |
| MOL004991 | 7-Acetoxy-2-methylisoflavone | 38.92 | 0.26 | Gancao |
| MOL004993 | 8-prenylated eriodictyol | 53.79 | 0.4 | Gancao |
| MOL004996 | gadelaidic acid | 30.7 | 0.2 | Gancao |
| MOL005000 | Gancaonin G | 60.44 | 0.39 | Gancao |
| MOL005001 | Gancaonin H | 50.1 | 0.78 | Gancao |
| MOL005003 | Licoagrocarpin | 58.81 | 0.58 | Gancao |
| MOL005007 | Glyasperins M | 72.67 | 0.59 | Gancao |
| MOL005008 | Glycyrrhiza flavonol A | 41.28 | 0.6 | Gancao |
| MOL005012 | Licoagroisoflavone | 57.28 | 0.49 | Gancao |
| MOL005013 | 18α-hydroxyglycyrrhetic acid | 41.16 | 0.71 | Gancao |
| MOL005016 | Odoratin | 49.95 | 0.3 | Gancao |
| MOL005017 | Phaseol | 78.77 | 0.58 | Gancao |
| MOL005018 | Xambioona | 54.85 | 0.87 | Gancao |
| MOL005020 | dehydroglyasperins C | 53.82 | 0.37 | Gancao |
| MOL000359 | sitosterol | 36.91 | 0.75 | Gancao,Huangqin |

**Abbreviations:** BCI, bioactive chemical ingredient; GQD, Gegen Qinlian decoction; OB, oral bioavailability; DL, drug-likeness.

**TABLE S2:** The significant GO-BP terms enriched by target genes.

| ID | Description | Adjusted  *P* value |
| --- | --- | --- |
| GO:0150077 | regulation of neuroinflammatory response | 1.89E-04 |
| GO:0007566 | embryo implantation | 1.89E-04 |
| GO:0150076 | neuroinflammatory response | 5.27E-04 |
| GO:0071241 | cellular response to inorganic substance | 5.27E-04 |
| GO:0042446 | hormone biosynthetic process | 5.27E-04 |
| GO:0007584 | response to nutrient | 5.45E-04 |
| GO:0042445 | hormone metabolic process | 5.45E-04 |
| GO:0001516 | prostaglandin biosynthetic process | 5.45E-04 |
| GO:0046457 | prostanoid biosynthetic process | 5.45E-04 |
| GO:0030574 | collagen catabolic process | 1.05E-03 |
| GO:0072593 | reactive oxygen species metabolic process | 1.05E-03 |
| GO:0033280 | response to vitamin D | 1.05E-03 |
| GO:0016999 | antibiotic metabolic process | 1.05E-03 |
| GO:0006692 | prostanoid metabolic process | 1.05E-03 |
| GO:0006693 | prostaglandin metabolic process | 1.05E-03 |
| GO:0046456 | icosanoid biosynthetic process | 2.03E-03 |
| GO:0010038 | response to metal ion | 2.03E-03 |
| GO:0006636 | unsaturated fatty acid biosynthetic process | 2.03E-03 |
| GO:0071248 | cellular response to metal ion | 2.12E-03 |
| GO:0048545 | response to steroid hormone | 3.00E-03 |
| GO:0001660 | fever generation | 3.01E-03 |
| GO:0031652 | positive regulation of heat generation | 3.01E-03 |
| GO:0007565 | female pregnancy | 3.01E-03 |
| GO:2000377 | regulation of reactive oxygen species metabolic process | 3.01E-03 |
| GO:0009612 | response to mechanical stimulus | 3.07E-03 |
| GO:0019371 | cyclooxygenase pathway | 3.07E-03 |
| GO:0031392 | regulation of prostaglandin biosynthetic process | 3.07E-03 |
| GO:1901224 | positive regulation of NIK/NF-kappaB signaling | 3.43E-03 |
| GO:0031650 | regulation of heat generation | 3.43E-03 |
| GO:0022617 | extracellular matrix disassembly | 3.56E-03 |
| GO:0030656 | regulation of vitamin metabolic process | 3.56E-03 |
| GO:0033127 | regulation of histone phosphorylation | 3.56E-03 |
| GO:2001279 | regulation of unsaturated fatty acid biosynthetic process | 3.56E-03 |
| GO:0044706 | multi-multicellular organism process | 3.73E-03 |
| GO:0006979 | response to oxidative stress | 3.73E-03 |
| GO:0070542 | response to fatty acid | 3.95E-03 |
| GO:0031667 | response to nutrient levels | 4.46E-03 |
| GO:0050900 | leukocyte migration | 4.46E-03 |
| GO:0031649 | heat generation | 4.62E-03 |
| GO:0033273 | response to vitamin | 4.94E-03 |
| GO:1901570 | fatty acid derivative biosynthetic process | 4.97E-03 |
| GO:1901652 | response to peptide | 5.04E-03 |
| GO:0033559 | unsaturated fatty acid metabolic process | 5.04E-03 |
| GO:0009991 | response to extracellular stimulus | 5.08E-03 |
| GO:1901615 | organic hydroxy compound metabolic process | 5.08E-03 |
| GO:2000379 | positive regulation of reactive oxygen species metabolic process | 5.08E-03 |
| GO:0006690 | icosanoid metabolic process | 5.08E-03 |
| GO:0032963 | collagen metabolic process | 5.08E-03 |
| GO:1990748 | cellular detoxification | 5.70E-03 |
| GO:0045723 | positive regulation of fatty acid biosynthetic process | 5.70E-03 |
| GO:0071498 | cellular response to fluid shear stress | 6.17E-03 |
| GO:0098754 | detoxification | 6.35E-03 |
| GO:1901222 | regulation of NIK/NF-kappaB signaling | 6.39E-03 |
| GO:0034754 | cellular hormone metabolic process | 6.77E-03 |
| GO:0046697 | decidualization | 7.39E-03 |
| GO:1903409 | reactive oxygen species biosynthetic process | 7.39E-03 |
| GO:0010818 | T cell chemotaxis | 7.73E-03 |
| GO:0032461 | positive regulation of protein oligomerization | 7.73E-03 |
| GO:0046683 | response to organophosphorus | 8.34E-03 |
| GO:0008202 | steroid metabolic process | 8.34E-03 |
| GO:0046885 | regulation of hormone biosynthetic process | 8.58E-03 |
| GO:0008209 | androgen metabolic process | 8.95E-03 |
| GO:1990776 | response to angiotensin | 8.95E-03 |
| GO:0030198 | extracellular matrix organization | 9.09E-03 |
| GO:0010575 | positive regulation of vascular endothelial growth factor production | 9.31E-03 |
| GO:0062013 | positive regulation of small molecule metabolic process | 9.74E-03 |
| GO:0006633 | fatty acid biosynthetic process | 9.74E-03 |
| GO:0014074 | response to purine-containing compound | 9.74E-03 |
| GO:0042133 | neurotransmitter metabolic process | 9.74E-03 |
| GO:0002675 | positive regulation of acute inflammatory response | 9.74E-03 |
| GO:0033198 | response to ATP | 9.74E-03 |
| GO:0071276 | cellular response to cadmium ion | 1.02E-02 |
| GO:1901568 | fatty acid derivative metabolic process | 1.12E-02 |
| GO:0010574 | regulation of vascular endothelial growth factor production | 1.13E-02 |
| GO:0001893 | maternal placenta development | 1.16E-02 |
| GO:0045923 | positive regulation of fatty acid metabolic process | 1.16E-02 |
| GO:0010573 | vascular endothelial growth factor production | 1.21E-02 |
| GO:0034405 | response to fluid shear stress | 1.26E-02 |
| GO:0034614 | cellular response to reactive oxygen species | 1.26E-02 |
| GO:0043062 | extracellular structure organization | 1.26E-02 |
| GO:0016572 | histone phosphorylation | 1.27E-02 |
| GO:0032350 | regulation of hormone metabolic process | 1.27E-02 |
| GO:1903034 | regulation of response to wounding | 1.36E-02 |
| GO:0019369 | arachidonic acid metabolic process | 1.36E-02 |
| GO:0051281 | positive regulation of release of sequestered calcium ion into cytosol | 1.36E-02 |
| GO:0010565 | regulation of cellular ketone metabolic process | 1.41E-02 |
| GO:0038061 | NIK/NF-kappaB signaling | 1.41E-02 |
| GO:0017001 | antibiotic catabolic process | 1.41E-02 |
| GO:0045429 | positive regulation of nitric oxide biosynthetic process | 1.41E-02 |
| GO:0048247 | lymphocyte chemotaxis | 1.41E-02 |
| GO:1904407 | positive regulation of nitric oxide metabolic process | 1.46E-02 |
| GO:0032459 | regulation of protein oligomerization | 1.52E-02 |
| GO:0006694 | steroid biosynthetic process | 1.61E-02 |
| GO:0006953 | acute-phase response | 1.67E-02 |
| GO:0042743 | hydrogen peroxide metabolic process | 1.67E-02 |
| GO:0062012 | regulation of small molecule metabolic process | 1.67E-02 |
| GO:0046890 | regulation of lipid biosynthetic process | 1.67E-02 |
| GO:0043281 | regulation of cysteine-type endopeptidase activity involved in apoptotic process | 1.68E-02 |
| GO:0034764 | positive regulation of transmembrane transport | 1.73E-02 |
| GO:0070098 | chemokine-mediated signaling pathway | 1.87E-02 |
| GO:0010524 | positive regulation of calcium ion transport into cytosol | 2.04E-02 |
| GO:0042304 | regulation of fatty acid biosynthetic process | 2.04E-02 |
| GO:1904645 | response to amyloid-beta | 2.04E-02 |
| GO:0050727 | regulation of inflammatory response | 2.14E-02 |
| GO:0071398 | cellular response to fatty acid | 2.15E-02 |
| GO:0046686 | response to cadmium ion | 2.17E-02 |
| GO:1903428 | positive regulation of reactive oxygen species biosynthetic process | 2.17E-02 |
| GO:2000116 | regulation of cysteine-type endopeptidase activity | 2.17E-02 |
| GO:2001234 | negative regulation of apoptotic signaling pathway | 2.18E-02 |
| GO:0032370 | positive regulation of lipid transport | 2.20E-02 |
| GO:0022411 | cellular component disassembly | 2.20E-02 |
| GO:0072678 | T cell migration | 2.20E-02 |
| GO:1901616 | organic hydroxy compound catabolic process | 2.20E-02 |
| GO:0046824 | positive regulation of nucleocytoplasmic transport | 2.20E-02 |
| GO:1990868 | response to chemokine | 2.20E-02 |
| GO:1990869 | cellular response to chemokine | 2.20E-02 |
| GO:0009636 | response to toxic substance | 2.20E-02 |
| GO:0000302 | response to reactive oxygen species | 2.20E-02 |
| GO:0060135 | maternal process involved in female pregnancy | 2.45E-02 |
| GO:0042180 | cellular ketone metabolic process | 2.45E-02 |
| GO:0097237 | cellular response to toxic substance | 2.45E-02 |
| GO:0045428 | regulation of nitric oxide biosynthetic process | 2.47E-02 |
| GO:0033002 | muscle cell proliferation | 2.47E-02 |
| GO:0051924 | regulation of calcium ion transport | 2.48E-02 |
| GO:0050805 | negative regulation of synaptic transmission | 2.65E-02 |
| GO:1904427 | positive regulation of calcium ion transmembrane transport | 2.78E-02 |
| GO:0043270 | positive regulation of ion transport | 3.09E-02 |
| GO:0006809 | nitric oxide biosynthetic process | 3.21E-02 |
| GO:0071260 | cellular response to mechanical stimulus | 3.27E-02 |
| GO:0034308 | primary alcohol metabolic process | 3.28E-02 |
| GO:0051279 | regulation of release of sequestered calcium ion into cytosol | 3.28E-02 |
| GO:1905954 | positive regulation of lipid localization | 3.28E-02 |
| GO:0043154 | negative regulation of cysteine-type endopeptidase activity involved in apoptotic process | 3.34E-02 |
| GO:0046209 | nitric oxide metabolic process | 3.45E-02 |
| GO:0046889 | positive regulation of lipid biosynthetic process | 3.45E-02 |
| GO:0051235 | maintenance of location | 3.54E-02 |
| GO:2001057 | reactive nitrogen species metabolic process | 3.65E-02 |
| GO:0032496 | response to lipopolysaccharide | 3.78E-02 |
| GO:0034599 | cellular response to oxidative stress | 3.78E-02 |
| GO:0072676 | lymphocyte migration | 3.78E-02 |
| GO:1903035 | negative regulation of response to wounding | 3.78E-02 |
| GO:2000117 | negative regulation of cysteine-type endopeptidase activity | 3.78E-02 |
| GO:0031058 | positive regulation of histone modification | 3.82E-02 |
| GO:0016042 | lipid catabolic process | 3.82E-02 |
| GO:0045833 | negative regulation of lipid metabolic process | 4.01E-02 |
| GO:1901655 | cellular response to ketone | 4.01E-02 |
| GO:0002237 | response to molecule of bacterial origin | 4.06E-02 |
| GO:0044070 | regulation of anion transport | 4.10E-02 |
| GO:1904062 | regulation of cation transmembrane transport | 4.10E-02 |
| GO:0001676 | long-chain fatty acid metabolic process | 4.10E-02 |
| GO:0007189 | adenylate cyclase-activating G protein-coupled receptor signaling pathway | 4.10E-02 |
| GO:2001243 | negative regulation of intrinsic apoptotic signaling pathway | 4.10E-02 |
| GO:0032103 | positive regulation of response to external stimulus | 4.11E-02 |
| GO:0019217 | regulation of fatty acid metabolic process | 4.11E-02 |
| GO:0050810 | regulation of steroid biosynthetic process | 4.11E-02 |
| GO:0072330 | monocarboxylic acid biosynthetic process | 4.14E-02 |
| GO:0018958 | phenol-containing compound metabolic process | 4.14E-02 |
| GO:0071496 | cellular response to external stimulus | 4.17E-02 |
| GO:0001505 | regulation of neurotransmitter levels | 4.17E-02 |
| GO:0009615 | response to virus | 4.17E-02 |
| GO:0010522 | regulation of calcium ion transport into cytosol | 4.17E-02 |
| GO:0042136 | neurotransmitter biosynthetic process | 4.17E-02 |
| GO:0098869 | cellular oxidant detoxification | 4.23E-02 |
| GO:0046822 | regulation of nucleocytoplasmic transport | 4.27E-02 |
| GO:0001101 | response to acid chemical | 4.27E-02 |
| GO:0048661 | positive regulation of smooth muscle cell proliferation | 4.27E-02 |
| GO:1903426 | regulation of reactive oxygen species biosynthetic process | 4.27E-02 |
| GO:1905269 | positive regulation of chromatin organization | 4.27E-02 |
| GO:0006066 | alcohol metabolic process | 4.54E-02 |
| GO:0006631 | fatty acid metabolic process | 4.54E-02 |
| GO:0042737 | drug catabolic process | 4.86E-02 |

**Abbreviations:** GO, Gene Ontology; BP, biological process.

**TABLE S3:** The significant GO-MF terms enriched by target genes.

| ID | Description | Adjusted  *P* value |
| --- | --- | --- |
| GO:0005125 | cytokine activity | 6.41E-05 |
| GO:0004252 | serine-type endopeptidase activity | 6.34E-04 |
| GO:0008236 | serine-type peptidase activity | 7.05E-04 |
| GO:0017171 | serine hydrolase activity | 7.05E-04 |
| GO:0045236 | CXCR chemokine receptor binding | 9.45E-04 |
| GO:0048018 | receptor ligand activity | 1.06E-03 |
| GO:0005126 | cytokine receptor binding | 1.08E-03 |
| GO:0030545 | receptor regulator activity | 1.09E-03 |
| GO:0016616 | oxidoreductase activity, acting on the CH-OH group of donors, NAD or NADP as acceptor | 2.35E-03 |
| GO:0016614 | oxidoreductase activity, acting on CH-OH group of donors | 2.88E-03 |
| GO:0004175 | endopeptidase activity | 3.44E-03 |
| GO:0033764 | steroid dehydrogenase activity, acting on the CH-OH group of donors, NAD or NADP as acceptor | 3.44E-03 |
| GO:0008009 | chemokine activity | 3.44E-03 |
| GO:0016229 | steroid dehydrogenase activity | 3.44E-03 |
| GO:0008237 | metallopeptidase activity | 3.90E-03 |
| GO:0004601 | peroxidase activity | 4.29E-03 |
| GO:0016684 | oxidoreductase activity, acting on peroxide as acceptor | 4.72E-03 |
| GO:0042379 | chemokine receptor binding | 6.14E-03 |
| GO:0016209 | antioxidant activity | 1.11E-02 |
| GO:0004222 | metalloendopeptidase activity | 1.94E-02 |
| GO:0016651 | oxidoreductase activity, acting on NAD(P)H | 2.15E-02 |
| GO:0020037 | heme binding | 2.48E-02 |
| GO:0005178 | integrin binding | 2.69E-02 |
| GO:0046906 | tetrapyrrole binding | 2.71E-02 |
| GO:0019887 | protein kinase regulator activity | 3.44E-02 |
| GO:0016705 | oxidoreductase activity, acting on paired donors, with incorporation or reduction of molecular oxygen | 3.44E-02 |
| GO:0052650 | NADP-retinol dehydrogenase activity | 3.47E-02 |
| GO:0008201 | heparin binding | 3.63E-02 |
| GO:0019207 | kinase regulator activity | 3.63E-02 |
| GO:0005149 | interleukin-1 receptor binding | 3.63E-02 |
| GO:0016863 | intramolecular oxidoreductase activity, transposing C=C bonds | 3.63E-02 |
| GO:0016641 | oxidoreductase activity, acting on the CH-NH2 group of donors, oxygen as acceptor | 3.80E-02 |
| GO:0004745 | retinol dehydrogenase activity | 3.97E-02 |
| GO:0004089 | carbonate dehydratase activity | 4.01E-02 |
| GO:0008106 | alcohol dehydrogenase (NADP+) activity | 4.01E-02 |
| GO:0050664 | oxidoreductase activity, acting on NAD(P)H, oxygen as acceptor | 4.16E-02 |
| GO:0016638 | oxidoreductase activity, acting on the CH-NH2 group of donors | 4.29E-02 |
| GO:0016538 | cyclin-dependent protein serine/threonine kinase regulator activity | 4.42E-02 |
| GO:0005539 | glycosaminoglycan binding | 4.64E-02 |
| GO:0001664 | G protein-coupled receptor binding | 4.78E-02 |

**Abbreviations:** GO, Gene Ontology; MF, molecular function.
